# Protective effects of Egyptian cloudy apple juice and apple peel extract on lipid peroxidation, antioxidant enzymes and inflammatory status in diabetic rat pancreas

**DOI:** 10.1186/s12906-015-0957-0

**Published:** 2016-01-11

**Authors:** Samah M. Fathy, Ehab A. Drees

**Affiliations:** 1 Zoology Department, Faculty of Science, Fayoum University, Fayoum, Egypt; 2 Biochemistry Division, Chemistry Department, Faculty of Science, Fayoum University, Fayoum, Egypt; 3 Biology Department, Faculty of Science, JazanUniversity, Jazan, Saudi Arabia

**Keywords:** Apple juice, Antioxidant enzymes, Apple peel, Diabetes, Inflammation, Lipid peroxidation

## Abstract

**Background:**

Apples possess rich content of varied polyphenolic compounds showing a variety of biological activities that may ascribe to worthy effects against some chronic diseases. The present study was designed to assess the protective effects of the cloudy apple juice (CAJ) and apple peel extract (APE) of Egyptian Anna apple on the complications in experimental diabetes.

**Materials and methods:**

Four groups were studied. Diabetes was induced by a single dose of streptozotocin (STZ) to only three groups of albino Wistar rats. Two of the diabetic groups received either CAJ or APE for 21 days. At the end of the study, lipid profile parameters were measured in serum while lipid peroxidation (LPO) level, antioxidant enzyme activities and inflammatory markers were evaluated in pancreas tissue samples. The gas chromatography–mass spectrometry (GC-MS) analysis of phenolic compounds found in CAJ and APE was carried out. Moreover, total phenolic content of CAJ and APE were measured.

**Results:**

The significant increase of blood glucose level, serum total cholesterol (TC), triglycerides (TG), low- density lipoprotein cholesterol (LDL-C), and very low-density lipoprotein (VLDL) levels, in addition to tissue malondialdehyde (MDA), nuclear factor kappa B (NF-kB), tumor necrosis factor α (TNF-α), interleukin 6 (IL-6), interleukin 8 (IL-8) levels, but a significant decrease in high-density lipoprotein cholesterol (HDL-C), and the activity of pancreatic antioxidant enzymes were the remarkably parameters observed in diabetic control rats. Dissimilarly, oral supplementation of 15 ml/kg CAJ and 1 g/kg APE for 21 days resulted in a significant decrease in fasting blood glucose, serum TC, TG, LDL-C, VLDL-C and tissue MDA, NF-kB, TNF-α, IL-6, IL-8 levels coupled with a significant elevation of HDL-C and antioxidant enzymes’ activity when compared with diabetic control animals.

**Conclusions:**

The results indicate that Egyptian CAJ and APE supplementation may have protective effects against deleterious complications of diabetes mellitus.

## Background

Diabetes mellitus (DM) is a major endocrine disorder and it is expected to increase worldwide by more than 100 % between 2000 and 2030 [1]. The effects of DM are devastating, which include the relative depletion of insulin secretion, hyperglycemia and altered metabolism of proteins, lipids and carbohydrates in addition to damage to the beta cells (β-cells) of pancreas and an increased risk of other serious complications of vascular diseases [2, 3]. Moreover, it was suggested that generation of free radicals is involved in the pathogenesis of diabetes, and the development of diabetic complications is due to the prolonged exposure to hyperglycemia that increases the formation of reactive oxygen species (ROS) via auto-oxidation of glucose and non-enzymatic protein glycation, which may cause disruption of cellular functions and oxidative damage to membranes. An elevation of ROS can cause an impairment of the antioxidant defense system or inability to scavenge the oxidative damage [4].

A correlation was detected between chronic low-grade inflammation and type 1 DM which is associated with a significant increase of serum biomarkers of inflammation such as IL-6 and TNF-α [5–8]. The leak out of various cytokines into the circulation of diabetic patients was observed [9]. These inflammatory cytokines were thought to be involved in the propagation of the disease and proposing a new role of inflammation via destruction of β-cells [9]. NF-kB is a critical transcription factor in inflammation, and its activation had been proven to upregulate gene expression of other pro-inflammatory cytokines [10]. It was suggested that NF-kB pathway regulates the production of the chemokine IL-8, which is one of the main mediators of inflammatory response [11]. Additionally, improper activation of NF-kB signaling pathway is associated with the appearance of many diseases including DM [11].

Streptozotocin (STZ) is a potent antibiotic, which is involved in the development of chemically-induced DM in experimental animals through its action on pancreatic β-cells [12]. The diabetogenic and cytotoxic effects of STZ are associated with the production of free radicals causing oxidative injury to the cells and also coupled with propagation of diabetes-linked autoimmunity [13–15]. High reactivity of ROS exerts toxic effects on the pancreatic acinar cells and is linked with inflammatory mediators’ production [16].

It has been reported that plasma thiobarbituric acid level increased in diabetic patients due to vascular lesions induced by hyperglycemia [17]. Lipid peroxidation was shown to be capable of initiating the secondary complications of diabetes. The level of ROS is modulated by the antioxidants, both enzymatic and non-enzymatic. Antioxidant enzymes such as superoxide dismutase (SOD), glutathione peroxidase (GPx), and catalase (CAT), while non-enzymatic antioxidants like reduced glutathione (GSH). In diabetes, oxidative stress results from the excessive generation of ROS, either by excess production or insufficient removal as a result of a sharp depletion of antioxidant potential [18].

Renewed attention to alternative medicines and natural therapies has animated new wave of research to look for more effective agents with lesser side effects [19]. Naturally occurring phytochemicals with antihyperglycemic activities are more favorable because they are commonly used to prevent morbidity and mortality from chronic diseases in countries with low or middle-income populations [20]. The widespread and increasing intake of apple juice/products and their rich phytochemical profile suggest their important potential to improve the health of populations consuming them [21]. Apples, the most consumed fruits of temperate climate countries, are a considerable source of phenolic compounds in the human diet [22]. Sun et al., showed that apples take the second position among fruits for the total phenolic compounds’ concentration [23]. Several studies have shown that apples have a wide variety of that may ascribe to health beneficial effects against cancer, cardiovascular diseases, asthma and pulmonary dysfunction, Alzheimer’s disease, decline of normal aging, weight management, bone health, diabetes and gastrointestinal protection [21]. In addition, apples have exhibited antioxidant and anti-inflammatory effects due to their phytochemicals and flavonoids contents [24, 25]. All these studies have supported the age-old saying “an apple a day keeps the doctor away”.

However, to the best of our knowledge, the present experimental study is a first-ever to analyze the Egyptian apple juice and peel phenolic profile and to evaluate their ameliorative potential on hyperglycemia, hyperlipidemia, pancreatic lipid peroxidation, pro-inflammatory cytokines, and the oxidative stress in STZ-induced diabetic animal model.

## Methods

### Experimental animals

Thirty two male albino Wistar rats (150–200 g) were housed in a temperature controlled room under 12/12 h light/dark cycle and had free access to pellet and tap water ad libitum.

This study was carried out in strict compliance with the Guidelines of Animals Health Research Institute, Egypt. The study protocol was approved by the Committee on the Ethics of Animals Health Research Institute, Egypt (Permit Number 362 approved on August 31, 2010). All the experimental procedures were carried out in accordance with NIH guide for the care and use of laboratory animals and with approval from the Local Animals Ethics Committee of NCI, Cairo University, Egypt.

### Apple source and identification

Egyptian Anna apple (*Malus domestica* Borkh) fruits were harvested at Nubaria city, Egypt. The plant was identified by matching it with well identified specimens kept at the Herbarium of Flora Researches Centre at the Agriculture museum campus (CAIM), Dokki, Giza, Egypt.

### Preparation of CAJ and APE

Ten kg of Anna apple (*Malus domestica* Borkh) fruits were washed and manually peeled. CAJ was obtained using commercial blender (Braun, Germany), and immediately stored at –20 °C for no longer than 2 months.

The APE was prepared according to Reagan-Shaw et al. [26]. The apples were washed in ddH_2_O, dried, and peeled using an autoclaved kitchen peeler then the peels were left to dry. Peels were homogenized at high speed with 2.5 ml ddH_2_O/g peel. The extract was centrifuged twice at 5000 g for 15 min and a third time at 10,000 g for 15 min. The supernatant was filter sterilized, aliquoted, and stored at –20 °C.

### Chemicals

Streptozotocin (STZ) was obtained from MP Biomedicals, LLC (lllkirch, France). All other chemicals used were of analytical grade.

### Induction of diabetes

The animals were fasted for 16 h prior to the induction of diabetes. STZ, freshly prepared in citrate buffer (0.1 M, pH 4.5), was administered intraperitoneally (i.p.) at a single dose of 60 mg/kg body weight. Development of diabetes was confirmed by polydipsia, polyuria and by determining glucose concentrations three days after injection of STZ. Rats with a blood glucose level of 250 mg/dl or above were considered to be diabetic. The treatment was initiated on the 4^th^ day after the injection of the STZ that was considered the first day of treatment and it was carried on continuously for 3 weeks.

### Experimental design

The rats were randomly assigned to 4 different treatment groups of 8 animals each. At the beginning of the experiment, each of the three diabetic groups received single dose of STZ in citrate buffer while the normal control group received citrate buffer only. The groups separated as indicated below.

Group I: normal control rats received citrate buffer (pH 4.5) (1 ml/kg. i.p.).

Group II: Diabetic control rats received a single dose of STZ (60 mg/kg. i.p.).

Group III: CAJ treated diabetic rats received CAJ (15 ml/kg/day; by gavage) on the 4^th^ day following STZ injection and continued for 21 days.

Group IV: APE treated diabetic rats received APE (1 g/kg/day; by gavage) on the 4^th^ day after STZ injection and continued for 21 days.

### Blood and tissue sampling

On the last day of the experiment, blood samples were collected for biochemical examinations. The samples were left for 30 min at room temperature and then centrifuged at 2000 g for 15 min at 4 °C for serum separation. The samples were kept frozen at –80 °C for the estimation of lipid profile parameters. Afterwards, the animals were decapitated and pancreas was immediately removed, rinsed in ice-cold phosphate buffered saline (0.1 M, pH 7.4) and weighed before homogenization. The minced tissue was homogenized in PBS and was sonicated. After that, the homogenates were centrifuged for 5 min at 5000 g at 4 °C to obtain the supernatant. Part of the supernatant was kept frozen at –80 °C for the estimation of MDA level and the activity of antioxidant enzymes; CAT, GR (glutathione reductase), GPx, and SOD while the other part was stored at ≤ –20 °C for determination of NF-kB, TNF-α, IL-6 and IL-8 levels.

### Gas chromatography–mass spectrometry (GC-MS) analysis

The analysis was carried out using a GC (Agilent Technologies 7890A) interfaced with a mass-selective detector (MSD, Agilent 7000 Triple Quad) equipped with an apolar Agilent HP-5 ms (5 %-phenyl methyl poly siloxane) capillary column (30 m × 0.25 mm i. d. and 0.25 μm film thickness). The carrier gas was helium with the linear velocity of 1 ml/min. The injector and detector temperatures were 200 °C and 250 °C, respectively. Injection mode, split; split ratio 1: 10, volume injected 1 μl of the sample. The MS operating parameters were as follows: ionization potential 70 eV, interface temperature 250 °C, and acquisition mass range 50–600. The identification of components was based on a comparison of their mass spectra and retention time with those of the authentic compounds and by computer matching with NIST and WILEY library as well as by comparison of the fragmentation pattern of the mass spectral data with those reported in the literature [27].

### Determination of the total phenolic content in CAJ and APE

The Folin–Ciocalteu method was used to determine total phenolic content [28]. Gallic acid was used as a standard and the determination was performed using spectrophotometer at 760 nm. All determinations were carried out in triplicate and results were expressed as percentage ± standard deviation (S.D.).

### Biochemical measurements

#### Blood glucose

Blood glucose was estimated using one touch glucometer (Accu check Roche, Germany).

#### Lipid profile parameters

Serum total cholesterol (TC) [29], total triglycerides (TG) [30], low-density lipoprotein cholesterol (LDL-C), very-low density lipoprotein cholesterol (VLDL-C) [31] and high-density lipoprotein cholesterol (HDL-C) [32] were determined by using standard colorimetric kits (Biosystems, Barcelona, Spain).

#### Estimation of pancreatic biochemical parameters

##### Measurement of MDA

Lipid peroxidation (LPO) in tissue homogenate was measured by thiobarbituric acid (TBA) reaction with MDA, a product formed due to the peroxidation of lipid membranes [33]. After a brief incubation, the samples were read spectrophotometically at 532 nm (OxiSelectTBARS Assay kit; MDA Quantitation kit, CellBiolabs, Inc., CA, USA).

##### GR assay

GR activity in tissue homogenate was assayed using a method based on GR reduces GSSG to GSH, which reacts with 5-(3-carboxy-4-nitrophenyl) disulfanyl-2-nitrobenzoic acid (DNTB) to generate TNB^2−^yellow color that was measured at 405 nm using a commercial available kit (BioVision, CA, USA) according to the manufacturer’s instructions.

##### CAT assay

CAT activity was measured using a CAT assay kit (Calbiochem, Germany) according to the manufacturer’s instructions. The method based on the reaction of the enzyme with methanol in the presence of an optimal concentration of H_2_O_2_. The formaldehyde produced was measured spectrophotometrically with 4-amino-3-hydrazino-5-mercapto-1,2,4-triazole (Purpald) as the chromagen [34]. Absorbance was read at 540 nm.

##### SOD assay

SOD activity was measured using OxiSelect SOD kit (CellBiolabs, Inc., CA, USA) according to the manufacturer’s instructions. The method based on the generation of superoxide anions (O_2_^−^) by a xanthine/xanthine Oxidase (XOD) systems, and then detected with a chromagen solution [35]. Absorbance was measured at 540 nm.

##### GPx assay

GPx activity was measured using Abnova kit (Taipei, Taiwan) according to the manufacturer’s instructions. In this assay, NADPH consumption was assessed by the enzyme coupled reactions [36]. The measured decrease in absorbance at 340 nm is directly proportional to the enzyme activity in the sample.

##### NF-kB, TNF-α, IL-6, and IL-8 measurement and ELISA

NF-kB, TNF-α, IL-6, and IL-8 levels were measured in the supernatant of the pancreatic tissue by enzyme-linked immunosorbent assay (ELISA) following the manufacturer’s instructions from (Millipore, CA.). One hundred microlitres of each supernatant was mixed with the assay buffer according to manufacturer’s instructions. The absorbance was determined spectrophotometrically at 450 nm ± 2 nm.

### Statistical analysis

Data were presented as means ± S. D. Data were analyzed by one-way analysis of variance (ANOVA) and significant differences were determined by Duncan’s multiple range test at 5 % level using statistical package for social science (SPSS 17, SPSS Inc. Chicago, IL, USA). Differences among groups were considered statistically significant at p < 0.05.

## Results

### GC-MS analysis

#### Polyphenolic compounds of CAJ

**Fig. 1:**
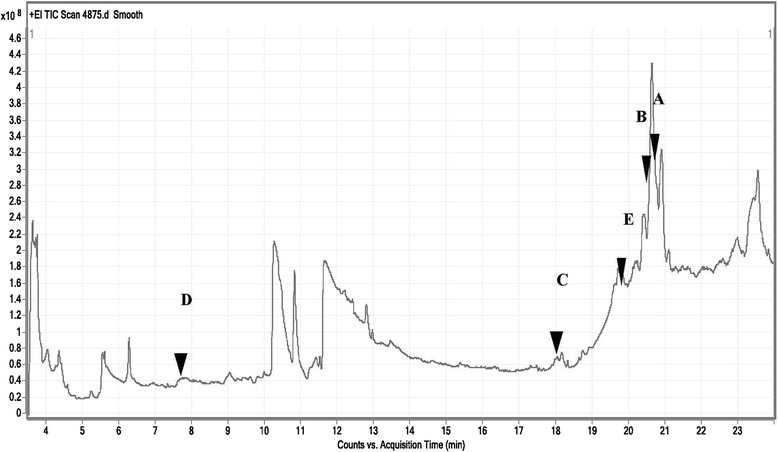
Total ion chromatograms of GC/MS analysis of Egyptian CAJ. **a**: Emodin (1,3,8-trihydroxy-6-methylanthracene-9,10-dione); **b**: Kaempferol (3,5,7-Trihydroxy-2-(4-hydroxyphenyl)-4H-chromen-4-one); **c**: Cyanidin cation [2-(3,4-dihydroxyphenyl)-3,5,7-trihydroxychromenylium]; **d**: Stevioside (13-[(2-O-beta-D-Glucopyranosyl-alpha-D-glucopyranosyl)oxy]kaur-16-en-18-oic acid beta-D-glucopyranosyl ester); **e**: Butylated hydroxytoluene (2,6-Bis(1,1-dimethylethyl)-4-methylphenol)

**Fig. 2:**
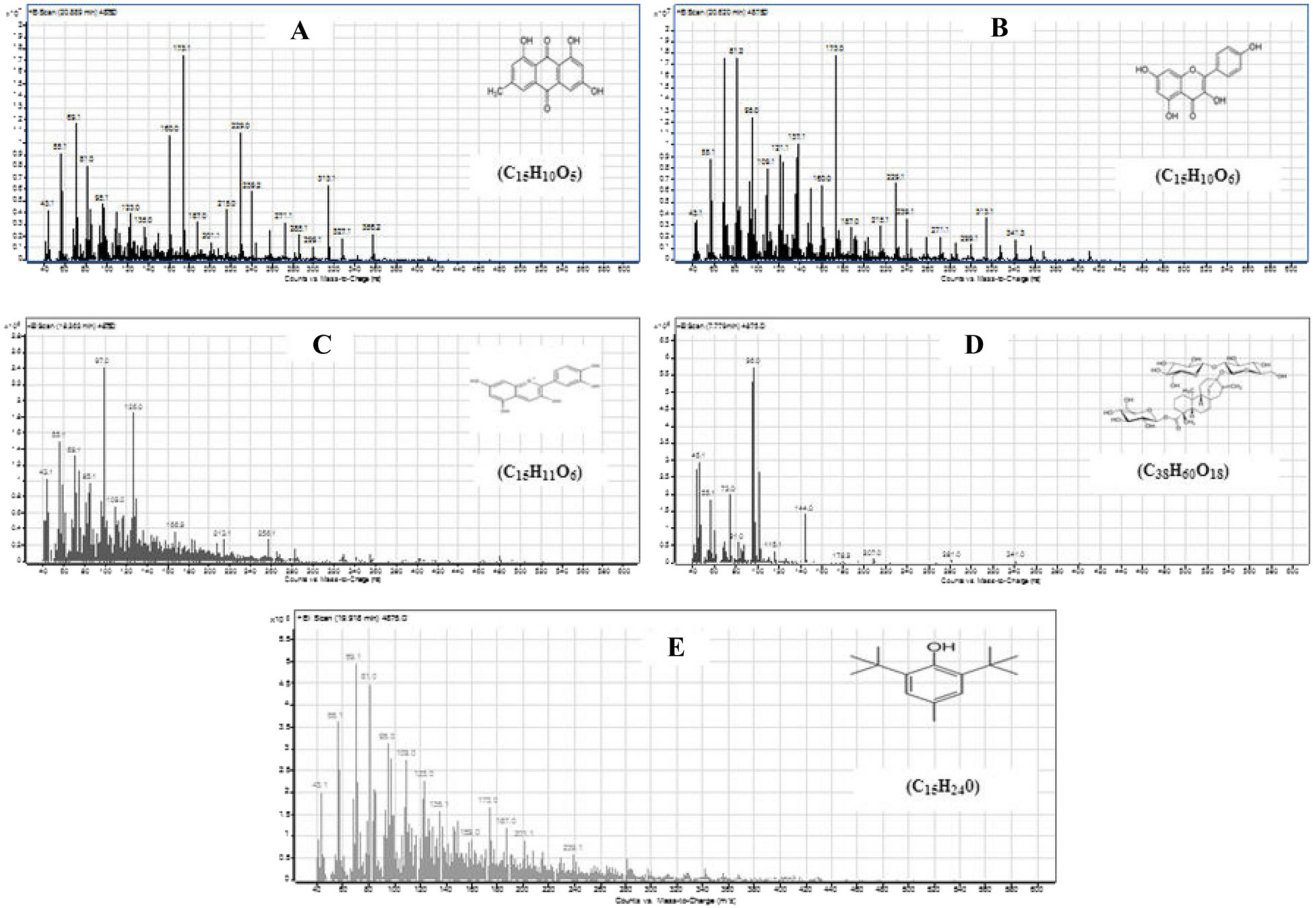
GC/MS fragmentation patterns of polyphenolic compounds in Egyptian CAJ. **a**: Emodin (1,3,8-trihydroxy-6-methylanthracene-9,10-dione); **b**: Kaempferol (3,5,7-Trihydroxy-2-(4-hydroxyphenyl)-4H-chromen-4-one); **c**: Cyanidin cation [2-(3,4-dihydroxyphenyl)-3,5,7-trihydroxychromenylium]; **d**: Stevioside (13-[(2-O-beta-D-Glucopyranosyl-alpha-D-glucopyranosyl)oxy]kaur-16-en-18-oic acid beta-D-glucopyranosyl ester); **e**: Butylated hydroxytoluene (2,6-Bis(1,1-dimethylethyl)-4-methylphenol)

GC-MS analysis of CAJ revealed the presence of different polyphenolic compounds. Those compounds include: Emodin (1,3,8-trihydroxy-6-methylanthracene-9,10-dione); Kaempferol (3,5,7-Trihydroxy-2-(4-hydroxyphenyl)-4H-chromen-4-one); Cyanidin cation [2-(3,4-dihdroxyphenyl)-3,5,7-trihydroxychromenylium]; Stevioside (13-[(2-O-beta-D-Glucopyranosyl-alpha-D-glucopyranosyl)oxy]kaur-16-en-18-oic acid beta-D-glucopyranosyl ester) and Butylated hydroxytoluene (2,6-Bis(1,1-dimethylethyl)-4-methylphenol) (Table 1, Figs. 1 and 2).

**Table 1:** GC-MS analysis of the phenolic compounds of CAJ

NO	Compounds	Retention time (min.)	Sum area %	Molar mass (gmol^−1^)
1	Emodin (1,3,8-trihydroxy-6-methylanthracene-9,10-dione)	20.88	33.78	270
2	Kaempferol (3,5,7-Trihydroxy-2-(4-hydroxyphenyl)-4H-chromen-4-one)	20.62	6.01	286
3	Cyanidin cation [2-(3,4-dihdroxyphenyl)-3,5,7-trihydroxychromenylium]	18.29	1.16	287
4	Stevioside (13-[(2-O-beta-D-Glucopyranosyl-alpha-D-glucopyranosyl)oxy]kaur-16-en-18-oic acid beta-D-glucopyranosyl ester)	7.77	1.63	804
5	Butylated hydroxytoluene (2,6-Bis(1,1-dimethylethyl)-4-methylphenol)	19.94	0.43	220

#### Polyphenolic compounds of APE

**Fig. 3:**
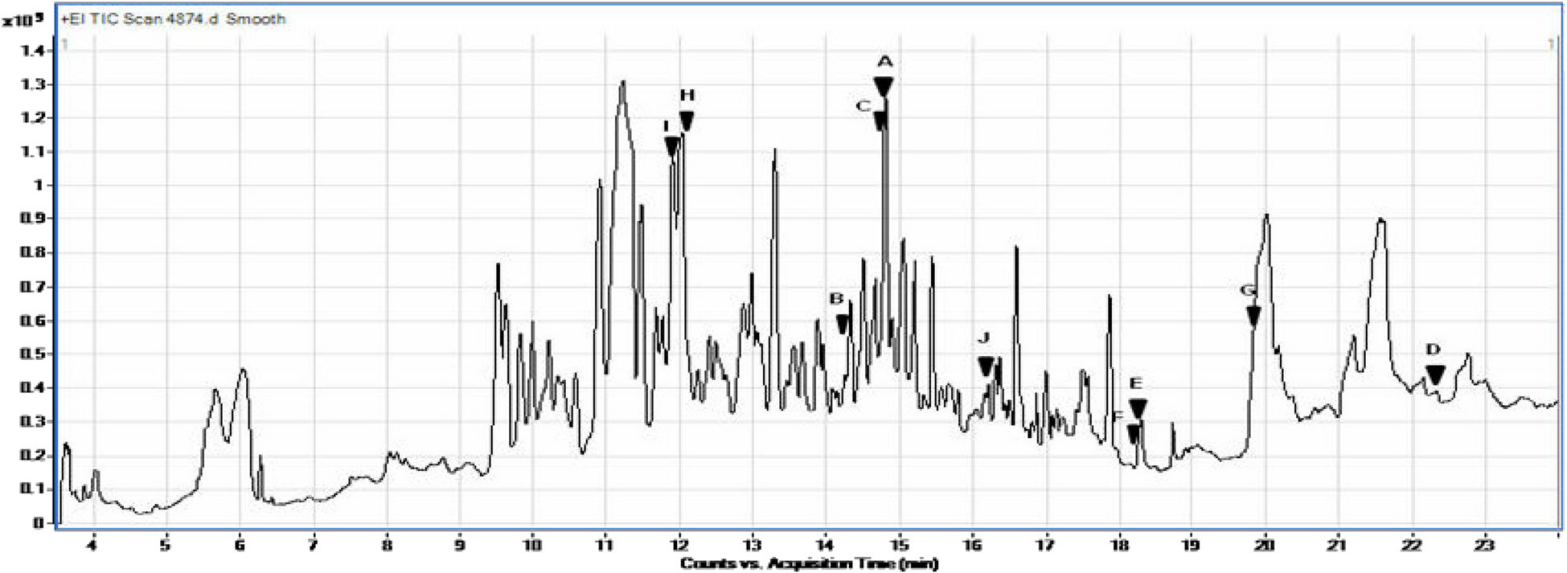
Total ion chromatograms of GC/MS analysis of Egyptian APE. **a**: (+)-Catechin [(2R,3S)-3′,4′,5,7-tetrahydroxyflavan-3-ol]; **b**: Resorcinol (Benzene-1,3-diol); **c**: Hydroquinone, 2,6-di-tert-butyl- [1,4 benzenediol, 2,6-bis (1,1 dimethylethyl)]; **d**: Resveratrol (3,5,4′-trihydroxy-trans-stilbene); **e**: Fisetin (3, 7, 3′, 4′-tetrahydroxyflavone); **f**: Cyanidin cation [2-(3,4-dihdroxyphenyl)-3,5,7 trihydroxychromenylium]; **g**: Butylated hydroxytoluene (2,6-Bis(1,1-dimethylethyl)-4-methylphenol); **h**: Patchoulene [1H-3a,7methanoazoulene, 2,3,6,7,8a hexahydro-1,4,9,9 tetramethyl-(1.alpha., 3a. alpha., 7. alpha., 8a. beta)]; **i**: Sinapic acid [3-(4-hydroxy-3,5-dimethoxyphenyl)prop-2-enoic acid]; **j**: Geranyl isovalerate (Trans-3,7 dimethyl-2,6-octadien-1-YL isopentanoate)

**Fig. 4:**
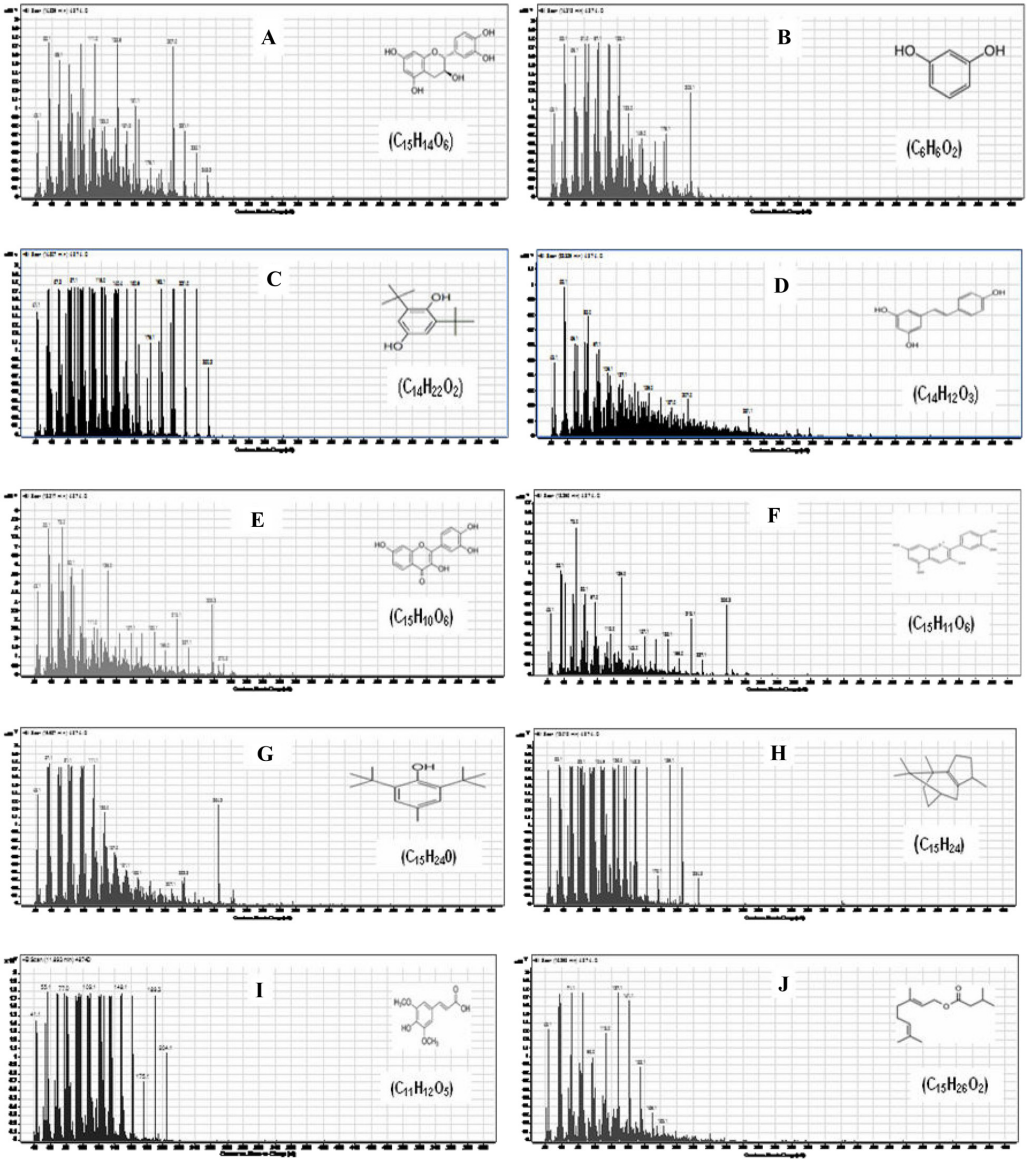
GC/MS fragmentation patterns of polyphenolic compounds in Egyptian APE. **a**: (+)-Catechin [(2R,3S)-3′,4′,5,7-tetrahydroxyflavan-3-ol]; **b**: Resorcinol (Benzene-1,3-diol); **c**: Hydroquinone, 2,6-di-tert-butyl- [1,4 benzenediol, 2,6-bis (1,1 dimethylethyl)]; **d**: Resveratrol (3,5,4′-trihydroxy-trans-stilbene); **e**: Fisetin (3, 7, 3′, 4′-tetrahydroxyflavone); **f**: Cyanidin cation [2-(3,4-dihdroxyphenyl)-3,5,7 trihydroxychromenylium]; **g**: Butylated hydroxytoluene (2,6-Bis(1,1-dimethylethyl)-4-methylphenol); **h**: Patchoulene [1H-3a,7methanoazoulene, 2,3,6,7,8a hexahydro-1,4,9,9 tetramethyl-(1.alpha., 3a. alpha., 7. alpha., 8a. beta)]; **i**: Sinapic acid [3-(4-hydroxy-3,5-dimethoxyphenyl)prop-2-enoic acid]; **j**: Geranyl isovalerate (Trans-3,7 dimethyl-2,6-octadien-1-YL isopentanoate)

GC-MS analysis of APE showed the presence of many various polyphenolic compounds. Those compounds include: (+)-Catechin [(2R,3S)-3′,4′,5,7-tetrahydroxyflavan-3-ol]; Resorcinol (Benzene-1,3-diol); Hydroquinone, 2,6-di-tert-butyl- [(1,4 benzenediol, 2,6-bis (1,1 dimethylethyl)]; Resveratrol (3,5,4′-trihydroxy-trans-stilbene); Fisetin (3, 7, 3′, 4′-tetrahydroxyflavone); Cyanidin cation [2-(3,4-dihdroxyphenyl)-3,5,7-trihydroxychromenylium]; Butylated hydroxytoluene (2,6-Bis(1,1-dimethylethyl)-4-methylphenol); Patchoulene [1H-3a,7methanoazoulene, 2,3,6,7,8a hexahydro-1,4,9,9 tetramethyl-(1.alpha., 3a. alpha., 7. alpha., 8a. beta)]; Sinapic acid [3-(4-hydroxy-3,5-dimethoxyphenyl)prop-2-enoic acid]; Geranyl isovalerate (Trans-3,7 dimethyl-2,6-octadien-1-YL isopentanoate) and β-Humulene (1,4,4-trimethyl-8-methylenecycloundeca-1,5-diene) (Table 2, Figs. 3 and 4).

**Table 2:** GC-MS analysis of the phenolic compounds of APE

NO	Compounds	Retention time (min.)	Sum area %	Molar mass (gmol^−1^)
1	(+)-Catechin [(2R,3S)-3′,4′,5,7-tetrahydroxyflavan-3-ol]	14.82	1.13	290
2	Resorcinol (Benzene-1,3-diol)	14.31	1.69	110
3	Hydroquinone, 2,6-di-tert-butyl- [1,4 benzenediol, 2,6-bis (1,1 dimethylethyl)]	14.8	2.31	222
4	Resveratrol (3,5,4′-trihydroxy-trans-stilbene)	22.3	0.5	228
5	Fisetin (3, 7, 3′, 4′-tetrahydroxyflavone)	18.31	0.56	286
6	Cyanidin cation [2-(3,4-dihdroxyphenyl)-3,5,7-trihydroxychromenylium]	18.29	1.21	287
7	Butylated hydroxytoluene (2,6-Bis(1,1-dimethylethyl)-4-methylphenol)	19.94	1.91	220
8	Patchoulene [1H-3a,7methanoazoulene, 2,3,6,7,8a hexahydro-1,4,9,9 tetramethyl-(1.alpha., 3a. alpha., 7. alpha., 8a. beta)]	12.01	5.99	204
9	Sinapic acid [3-(4-hydroxy-3,5-dimethoxyphenyl)prop-2-enoic acid]	11.89	3.89	224
10	Geranyl isovalerate (Trans-3,7 dimethyl-2,6-octadien-1-YL isopentanoate)	16.29	0.73	238
11	β-Humulene (1,4,4-trimethyl-8-methylenecycloundeca-1,5-diene)	11.43	4.81	204

### Total phenolic contents in CAJ and APE

It was found that the total amount of phenolic compounds in CAJ are 69.68 ± 2.17 mg of gallic acid equivalents/100 ml of juice whereas the total phenolic content in APE are 673.46 ± 6.90 mg of gallic acid equivalents/100 g DW.

### Effect of CAJ and APE supplementation on hyperglycemia in experimental animals

The levels of blood glucose in normal and diabetic rats are presented in Table 3. In all groups prior to STZ injection, the basal level of blood glucose of the rats was not markedly different. However, there was a significant elevation in blood glucose level after STZ injection. Although a significant antihyperglycemic effect was obvious from the first week onwards, the reduction in blood glucose was maximum on the third week in diabetic group receiving CAJ. After STZ injection, a significant increase in blood glucose was observed in diabetic control rats as compared with normal control rats (*P* < 0.05). The administration of CAJ and APE significantly decreased glucose levels in diabetic rats when compared with diabetic control group (*P* < 0.05). Moreover, CAJ and APE treated diabetic groups exhibited insignificant differences when compared with normal control rats. Therefore, the levels of blood glucose reverted close to normal range in diabetic rats treated with CAJ and APE.

**Table 3:** Effect of CAJ and APE supplementation for 21 days on blood glucose levels in experimental animals

Groups	Blood glucose (mg/dl)				
	0 day	3 days after STZ	First week	Second week	Third week
Normal control (NC)	84.23 ± 9.24		89.45 ± 8.12**	89.57 ± 13.99**	81.53 ± 8.62**
Diabetic control (DC)	85.07 ± 5.90	339.01 ± 7.36	374.67 ± 9.87*	408.25 ± 10.50*	432.14 ± 10.31*
Diabetic + CAJ (15 ml/kg)	82.67 ± 8.19	355.77 ± 12.33	134.83 ± 8.19**	99.40 ± 14.33**	87.87 ± 9.64**
Diabetic + APE (1 g/kg)	81.57 ± 8.75	399.23 ± 14.92	90.67 ± 14.30**	88.0 ± 5.23**	105.72 ± 36.10**

The data are expressed as mean ± S.D. of 8 rats in each group

Average in the same column having different superscripts (*, **) indicate significant differences between the values (*P* <0.05)

### Effect of CAJ and APE supplementation on hyperlipidemia in experimental animals

Table 4 indicates the serum levels of lipid TC, TG, HDL-C, LDL-C and VLDL-C in normal and experimental animals. STZ injection led to a significant elevation of TC, TG, LDL-C, VLDL-C and reduction in HDL-C levels when compared to the normal control rats (P < 0.05). There was a significant reduction in TC, TG, LDL-C, VLDL-C in diabetic rats treated with CAJ and APE as compared with diabetic control rats (P < 0.05). A pronounced elevation of HDL-C in diabetic groups receiving CAJ and APE, as compared with diabetic control rats, was recorded (P < 0.05). Notably, all lipid profile parameters were reversed close to normal control levels following CAJ and APE supplementations as the differences were insignificant among CAJ treated diabetic, APE treated diabetic and normal control groups.

**Table 4:** Effect of CAJ and APE supplementation for 21 days on serum lipid profile in experimental animals

Groups	TC (mg/dl)	TG (mg/dl)	HDL-C (mg/dl)	LDL-C (mg/dl)	VLDL (mg/dl)
Normal Control (NC)	97.77 ± 20.58**	87.76 ± 23.41**	47.86 ± 4.33**	54.89 ± 2.60**	16.15 ± 5.09**
Diabetic Control (DC)	219.79 ± 2.60*	134.91 ± 32.46*	33.91 ± 6.19*	167.61 ± 8.51*	24.89 ± 5.25*
Diabetic + CAJ (15 ml/kg)	87.93 ± 14.77**	75.41 ± 33.79**	49.01 ± 7.03**	43.27 ± 1.80**	12.48 ± 5.71**
Diabetic + APE (1g/kg)	108.68 ± 14.20**	69.40 ± 21.90**	46.95 ± 8.13**	66.03 ± 3.00**	14.49 ± 4.65**

The data are expressed as mean ± S.D. of 8 rats in each group

Average in the same column having different superscripts (*, **) indicate significant differences between the values (*P* <0.05)

### Effect of CAJ and APE supplementation on pancreatic lipid peroxidation (LPO) in experimental animals

Table 5 shows the effect of CAJ and APE administration on MDA levels in pancreatic tissue of diabetic rats. There was a significant elevation in tissue MDA in diabetic rats as compared with normal control rats (*P* < 0.05). Treatment of diabetic rats with CAJ and APE for 21 days results in a marked decrease in pancreatic MDA when compared with diabetic control rats (P < 0.05). Meanwhile, the differences were significant between both CAJ and APE treated diabetic rats in relation to normal control group (*P* < 0.05).

**Table 5:** Effect of CAJ and APE supplementation for 21 days on lipid peroxidation (MDA) levels and antioxidant enzyme activities in pancreas of diabetic rats

Groups	MDA	CAT	GR	GPx	SOD
	(μmol/10 mg protein)	(nmol/min/10 mg protein)	(mU/10 mg protein)	(U/10 mg protein)	(U/10 mg protein)
Normal Control (NC)	16.94 ± 1.25**	38.09 ± 1.79**	24.47 ± 1.45**	7.16 ± 0.1**	11.21 ± 0.97**
Diabetic Control (DC)	27.08 ± 1.20*	27.36 ± 1.48*	17.79 ± 0.46*	3.54 ± 0.64*	7.23 ± 0.30*
Diabetic + CAJ (15 ml/kg)	20.73 ± 1.35***	34.35 ± 0.76****	23.75 ± 1.34**	6.26 ± 0.12****	9.77 ± 0.16****
Diabetic + APE (1g/kg)	20.45 ± 0.75***	29.59 ± 1.09***	20.93 ± 0.77***	4.92 ± 0.19***	8.26 ± 0.63***

The data are expressed as mean ± S.D. of 8 rats in each group

Average in the same column having different superscripts (*, **, ***, ****) indicate significant differences between the values (*P* <0.05)

### Effect of CAJ and APE supplementation on pancreatic antioxidant enzymes activities in experimental animals

As presented in Table 5, the activities of enzymatic antioxidants (CAT, GR, GPx and SOD) in pancreas of normal control, diabetic control and diabetic treated rats. CAT, GR, GPx and SOD activities were significantly depleted in diabetic control rats as compared with normal control rats (P < 0.05). A significant improvement in the activities of these enzymes after oral administration of CAJ and APE, when compared with diabetic control rats, was recorded (P < 0.05). Regarding CAT, GPx and SOD activities, significant variations were observed among diabetic control, CAJ treated diabetic and APE treated diabetic groups (P < 0.05). However, GR activity showed a significant difference between APE treated diabetic group compared to either normal control group or CAJ treated diabetic rats (P < 0.05).

### CAJ and APE attenuated the production of NF-kB, TNF-α, IL-6, and IL-8in diabetic rats

**Fig. 5:**
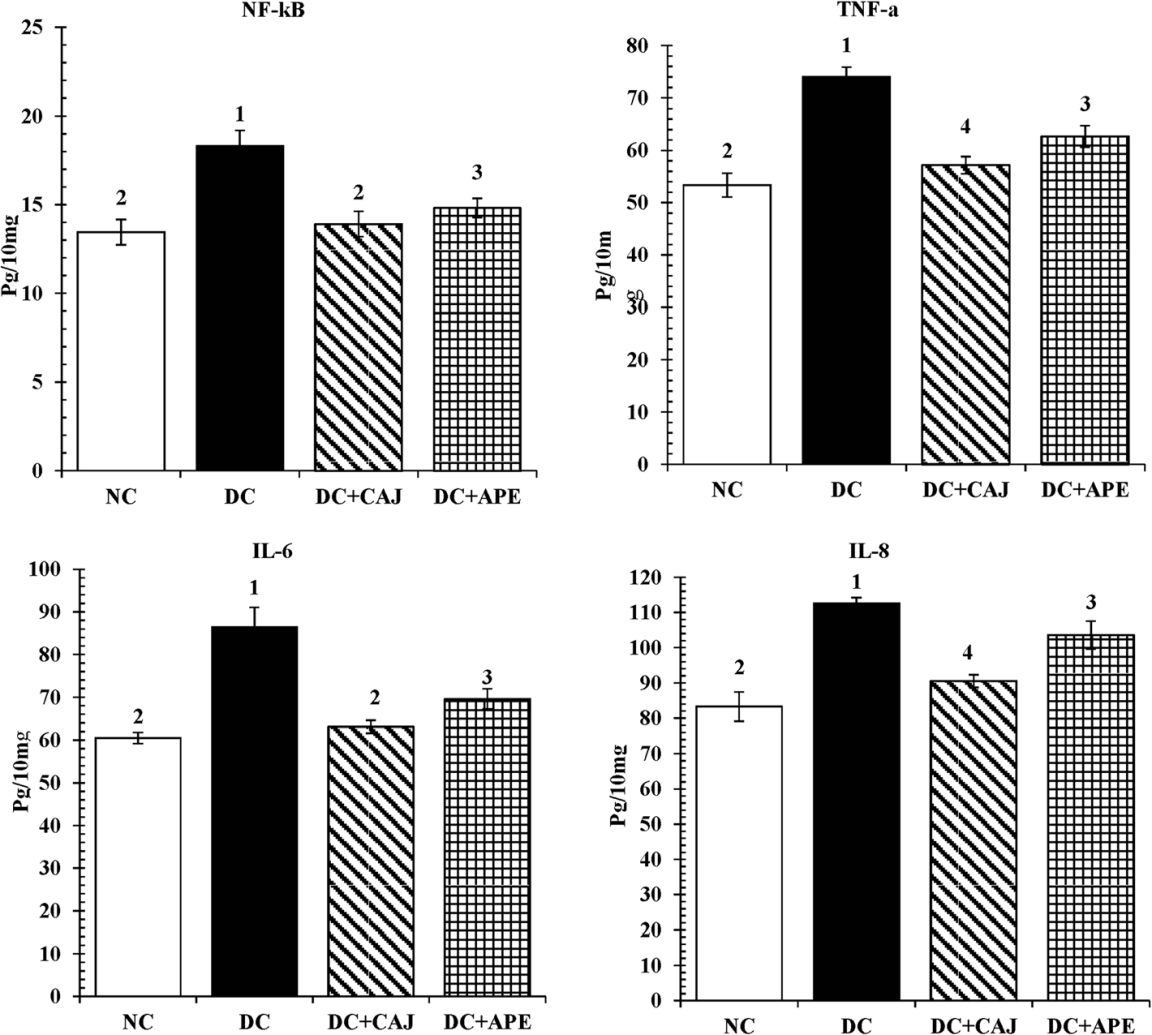
The effect of CAJ and APE for 21 days on pancreatic NF-kB, TNF-α, IL-6, and IL-8. Data are expressed as mean ± S.D. of 8 rats in each group. (NC = normal control group; DC = diabetic control group; DC + CAJ = diabetic group treated with CAJ; DC + APE = STZ-diabetic group treated with APE. Average in the same parameter having different superscripts (1, 2, 3, 4) indicate significant differences between the values (*P* <0.05)

Figure 5 revealed that STZ significantly increased the production of the pancreatic pro-inflammatory markers; NF-kB, TNF-α, IL-6, and IL-8 as compared with the normal control rats (P < 0.05). Meanwhile, treatment with CAJ and APE significantly reduced the levels of those inflammatory mediators when compared with diabetic control group (P < 0.05).

Regarding the level of NF-kB, diabetic control group showed a significant elevated NF-kB level in relation to normal control rats (P < 0.05); whereas the treatment with both CAJ and APE significantly reduced NF-kB levels as compared to diabetic control group (P < 0.05). Although CAJ treatment reversed NF-kB level towards normal control level, a significant difference in the level of NF-kB was recorded between APE supplemented diabetic group when compared with normal control rats (P < 0.05).

In addition, TNF-α level was significantly enhanced in diabetic control rats in relation to normal control group (P < 0.05); while CAJ and APE supplementations reduced the levels of TNF-α as compared with diabetic control group (P < 0.05). Moreover, the difference between either CAJ or APE treated diabetic groups was significant in relation to normal control group (P < 0.05).

Regarding the level of IL-6, it was found that the diabetic control group have a significantly elevated level when compared with normal control level (P < 0.05). Meanwhile the treatment of diabetic rats with either CAJ or APE significantly reduced IL-6 levels as compared to diabetic control rats (P < 0.05) and ameliorated the IL-6 levels close to normal control group. However, a significant difference was detected between APE treated diabetic group as compared with both normal control and CAJ treated diabetic rats (P < 0.05).

On the other hand; the level of IL-8 diabetic control group showed a highly significant increase in comparison with normal control group (P < 0.05); whereas, IL-8 level was decreased with CAJ and APE treatments compared to diabetic control group (P < 0.05). Notably, significant differences among IL-8 levels of CAJ treated diabetic, APE treated diabetic and normal control groups were detected (P < 0.05).

## Discussion

Several studies have addressed the link between dietary fruit and vegetables’ consumption in relation to improved health in human. Fruit and vegetables are precious source of many biologically active components including antioxidants, which play a key role as chemopreventive agents and possessing a clear effect on retarding the development of serious chronic diseases’ complications [37]. Apples are known to be rich in flavonoid antioxidants including epicatechins, catechins, and procyanidins which are the major phytochemicals in the human diet [38]. Although apples and apple products have been shown to exert positive effects in numerous pathologies [39], the preventive activity of Egyptian Anna apple against chemically induced diabetes has not been explored.

In the current work, supplementation of CAJ and APE for 21 days shows antihyperglycemic effect in STZ-induced diabetic rats. Such results are in agreement with previous reports that proved antihyperglycemic effects of pomegranate, black tea, and green tea in chemically induced diabetic animals [40–42].

The present polyphenolic investigation of CAJ and APE demonstrated the finding of bioflavonoids that may have a role in the stimulation of glucose uptake in peripheral tissues and regulation of the expression and/or activity of the enzymatic committed steps involved in carbohydrate metabolism [43]. Further study is required to estimate the impact of CAJ and APE on the activity and/or expression of such enzymes.

Hyperlipidemia is a recognized consequence of DM and also is one of the major cardiovascular risk factors. Diabetes induced hyperlipidemia is ascribed to a variety of derangement in regulatory and metabolic processes which coming from the excess mobilization of fats from adipocytes due to less utilization of glucose caused by insulin deficiency, which consequently leads to accumulation of lipids such as TC and TG in diabetic patients [44]. Our data were in agreement with previous observation that STZ-induced diabetic rats showed a significant elevation of TC, TG, LDL-C, and VLDL-C levels in addition to a reduction in HDL-C level. It is known that HDL participates in the transport of cholesterol from peripheral tissues to the liver, thus inhibiting the genesis of atherosclerosis [45]. Treatment with CAJ and APE for 21 days was sufficient to produce a significant decrease in serum TC, TG, LDL-C and VLDL-C levels suggesting that the polyphenolic components of these apple products (CAJ & APE) have a hypolipidemic effect. The marked elevation in the level of HDL suggests a cardio-protective effect of CAJ and APE treatment. In support for our results, hypolipidimic impact of different polyphenolic compounds was recorded in *Hibiscus sabdariffa* plant [46].

Oxygen free radicals have been suggested to ascribe to the development of complications of diabetes leading to the cytotoxicity of β-cells. Indeed, several studies have been carried out to evaluate changes in parameters of oxidative stress in diabetes [47]. In the present study, an increase in MDA in the pancreas of STZ-induced rats was observed when compared with normal control animals. MDA serves as an index of LPO [48]. Subsequently, increased MDA level was in agreement with the results of a previous study [40]. Thus, the increased MDA level in DM suggests that the hyperglycemia induces the peroxidative reactions in lipids [49]. Supplementation of CAJ and APE potentially reduced MDA level, suggesting that CAJ and APE polyphenolic constituents might have antioxidant properties to counteract MDA elevation and protect the pancreas from diabetic oxidative stress. This result is in consistent with Ramos who attributed MDA depletion and oxidative stress rescue effects in cancer to the presence of dietary polyphenols [50]. This is further supported by evidence revealing the use of natural extracts from plant source in rendering the risk of oxidative stress due to their rich source of phytochemicals [51].

An important consequence of diabetic hyperglycemia is the enhanced oxidative stress resulting from an imbalance between production and neutralization of ROS by antioxidant defense system. This imbalance impairs the cellular functions leading to various pathological conditions and also an alteration in the activity of antioxidant enzymes, such as CAT, GR, GPx, SOD and disturbed reduced glutathione [52].

The present data showed that STZ-induced diabetes impairs actions of pancreatic antioxidant enzymes. The significant decline in the activities of these enzymes may be attributed to the accumulation of ROS such as hydrogen peroxide (H_2_O_2_), superoxide (O_2_) and hydroxyl (OH^−^) [53, 54], which would have otherwise been effectively scavenged by antioxidant enzymes. CAJ and APE supplementations were significantly potentiated the pancreatic antioxidative defense and elevated the activities of these enzymes in diabetic rats (Table 5). This result is in agreement with Avci et al. who reported that apple consumption increased antioxidant enzymes, including SOD and GPx in erythrocytes and overall antioxidant potential in the plasma [55].

It has been found that there is no harmony in the level of antioxidant enzymes of many organs in diabetic rats. Although, some studies determining activities of CAT and SOD in DM exhibited the depletion in the activity of these enzymes [56, 57], other studies reported the increment in the activity of both enzymes with the STZ-induced diabetes [58, 59]. SOD is considered a first-line defense against ROS. This enzyme is present in nearly all cells, and scavenges the superoxide radicals by converting it to H_2_O_2_ [60]. In the present study, the activity of SOD was found to be lower in pancreatic tissues of control diabetic rats. The marked depletion in SOD activity could be attributed to accumulation of H_2_O_2_ or by glycation of the enzyme which have been reported to occur in diabetic state [61]. Further CAT, a peroxidase enzyme, is the most important enzyme involved in the detoxification of H_2_O_2_. The reduction in CAT activity in control diabetic pancreas tissue could result from glycation of the enzyme or due to chronic exposure to H_2_O_2_ in vivo [62]. H_2_O_2_ may be an essential mediator for any possible damage in STZ-induced diabetes [63]. The same pattern of results was found for the decreased activity of GPx. This enzyme catalyzes the reduction of hydroperoxides at the expense of reduced glutathione [64]. In the current study, the significantly depletion in pancreatic CAT, GR, GPx and SOD activities could be attributed to excessively produced free radicals, which crosses the scavenging potency of these enzymatic antioxidants in diabetic control rats. Treatment with CAJ and APE for 21 days, the levels of these pancreatic antioxidant enzymes were significantly augmented in diabetic rats which could be attributed to the antioxidative properties of polyphenolic compounds that were found in CAJ and APE. The current polyphenolic analysis of CAJ and APE showed the existence of bioflavonoids that may exhibit a strong free radical-scavenging activity. Such antioxidant properties of polyphenols have been intensively reported [16, 65] and could be ascribed to not only by the scavenging of free radicals, but also by the modulation of the mitochondrial function which represents the major cellular source of ROS [66].

DM propagation is associated with a chain of molecular events including inflammation which was proven to be a key factor in this disease [67]. It has been reported that type 1 DM is an autoimmune disease that is characterized by dysfunction and damage of pancreatic β-cells which is a consequence of direct contact with immune cells and of exposure to cytotoxic pro-inflammatory cytokines as well as other toxic substances [68]. Consequently, the regulation of the intra pancreatic inflammatory markers is an attractive avenue in DM treatment.

NF-kB is considered as a critical key target for alleviation of inflammation and a series of molecular cascades can be induced by its modulation [69]. NF-kB activity is induced in response to various cellular physiological stresses such as DM followed by NF-kB target genes expression [69]. The current study showed elevated activity of pancreatic NF-kB after STZ injection in relation to control samples. This pro-inflammatory response might be attributed to oxidative stress [70] via ROS production [16]. It was shown that excessive production of ROS in adipocytes disturbs endoplasmic reticulum (ER), alters mitochondrial function and induces inflammatory reaction [71, 72]. ER stress-mediated NF-kB activation was proven to be dependent on efflux of calcium from ER followed by production of reactive oxygen intermediates (ROI) [16].

NF-kB pathway controls the release of both IL-6 and IL-8 that are involved in DM type 1 development [11]. In addition, it was demonstrated that both TNF-α and IL-6 are target genes for NF-kB [73, 74]. In the present experiment, STZ increased the levels of pro-inflammatory mediators; TNF-α, IL-6 and IL-8. It has been reported that the levels of pancreatic TNF- α, IL-6 and IL-8 inflammatory cytokines were augmented in STZ-induced diabetic rats [75, 76]. For further support, IL-6 and TNF-α showed elevation of their levels in the pancreatic islets of STZ-induced type 1 rats [77, 78]. TNF-α is an important inflammatory mediator which is induced by ROS [16]. It has been reported that TNF-α has a role in the production of IL-6 via NF-kB signaling pathway activation [16].

Supplementation of CAJ and APE ameliorated the levels of inflammatory cytokines in STZ-induced diabetic rats. The beneficial impact of CAJ and APE treatments might be due to anti-inflammatory impact of the recently explored polyphenols of CAJ and APE through blocking of oxidative stress and mitochondrial damage [79] as well as free radical scavenging activity leading to antioxidant enzymes enhancement [80]. In addition, the suppression of NF-kB activity modulates antioxidant impact which might in turn inhibit the expression of inflammatory cytokines [81].

The results of current comprehensive study provide reasonable information on the Egyptian CAJ and APE polyphenols. The GC-MS analysis delivers the composition of the different biomolecules in the Egyptian apple (CAJ and APE).

Polyphenols are divided into diverse groups according to the number of phenol rings and the chemical groups attached to these rings. Flavonoids constitute the most important group of polyphenols that contain compounds such as catechin, kaempferol and cyanidine [27]. The previous components were exhibited in the current work either in CAJ or APE or both (Tables 1, 2 and Figs. 1, 2, 3, 4). Moreover, other polyphenolic compounds were recorded by the GC-MS analysis such as emodin and steviosides in CAJ, pachoulene, sinapic acid, fisetin, resorcinol, and resveratrol in APE. In addition, Triterpenoids including; β-Humulene and Geranyl isovalerate were detected.

Collectively, the currently detected constituents of the Egyptian CAJ and APE polyphenols and phytochemicals have been reported to intimately exert antihyperglycemic, antihyperlipidemic, antioxidative and anti-inflammatory consequences. Catechin is one of the flavonoids that is proven to effectively reduce lipid peroxidation and scavenge free radicals [82, 83]. Kaemferol possess antioxidative and hypolipidemic properties [79]. Cyanidine is one of the naturally occurring anthocyanin that acts as antioxidant via scavenging ROS or reactive nitrogen species (RNS), reducing specific enzymes, chelating trace metals associated with the free radical production inducing antioxidant defense, and breaking the free-radical chain reaction [84, 85]. Emodin treatment significantly reduced NF-kB DNA binding activity and the serum expression levels of TNF-α, IL-6 which led to reduced pancreatic MDA and increased SOD levels in severe acute pancreatitis rat model [81]. Stevioside is a natural sweetener that is proven to exert antihyperglycemic effect due to its capability of scavenging reactive oxygen species (ROS) and improving antioxidant response [86]. Fisetin possesses antioxidative, antihyperglycemic and anti-inflammatory properties [79]. Sinapic acid markedly reduced lipid peroxidation and depleted the activation of NF-kB in dimethylnitrosamine-induced fibrosis in rats [87].

As shown in Tables 1 and 2 as well as in Figs. 1, 2, 3 and 4, all the previous elements are the major phenolic compounds that are determined in either CAJ and/or APE. The antihyperglycemic impacts of the Egyptian CAJ or APE may be contributed by these polyphenolic components through their antioxidant strengths and scavenging activities, to restore the antioxidant enzymes in the pancreas, repair disoriented lipid profile and suppress the intra-pancreatic levels of pro-inflammatory mediators.

## Conclusions

From the above findings, we conclude that Egyptian Anna CAJ and APE possess antihyperglycemic effects by reduction of the inflammatory response, mitigation of the oxidative stress, and normalization of the deranged lipid profile. Thus, Egyptian CAJ and APE may be useful therapeutic agents that protect from DM.
